# Reviewer Acknowledgment

**DOI:** 10.1002/1348-9585.12433

**Published:** 2023-11-17

The Japan Society for Occupational Health expresses its sincere appreciation to the following volunteers for reviewing manuscripts for *Journal of Occupational Health* from October 1, 2022 to September 28, 2023.

Adi, Nuri Purwito (Japan)

Anderson, Amanda (United States)

Ando, Hajime (Japan)

Antao, Helena Sofia (Portugal)

Arakawa, Ritsuko (Japan)

Aung, Myo (Japan)

Azuma, Kenichi (Japan)

Bazazan, Ahmad (Iran)

Cheng, Wan‐Ju (China)

Cheng, Yawen (Taiwan)

Choi, Byungjoo (Korea, the Republic of)

Cruz, Alex Junio Silva (Brazil)

Deepreecha, Kathawoot (Thailand)

Del Razo, Luz María (Mexico)

Dollard, Maureen (Australia)

Dragano, Nico (Germany)

Du, Tanghuizi (Japan)

Dugdale, Zoe (United States)

Ebara, Takeshi (Japan)

Eguchi, Hisashi (Japan)

Eguchi, Yasumasa (Japan)

Ely Zarina, Samsudin (Malaysia)

Ernawati, Ernawati (Indonesia)

Fink, Anne (United States)

Formazin, Maren (Germany)

Franke, Warren (United States)

Fujino, Yoshihisa (Japan)

Fujiyoshi, Akira (Japan)

Fukai, Kota (Japan)

Fukushima, Noritoshi (Japan)

Fukutani, Naoto (Japan)

Furuta, Michiko (Japan)

Fushimi, Atsushi (Japan)

Gi, Min (Japan)

Gillespie, Gordon L (United States)

Hanners, Audra (United States)

Hara, Kunio (Japan)

Hara, Megumi (Japan)

Harada, Arisa (Japan)

Harmer, Bonnie (United States)

He, Yupeng (Japan)

Hidaka, Tomoo (Japan)

Higashi, Hidenori (Japan)

Higuchi, Yoshiyuki (Japan)

Hikichi, Hiroyuki (Japan)

Hino, Ayako (Japan)

Hiraku, Yusuke (Japan)

Hirokawa, Kumi (Japan)

Honda, Takanori (Japan)

Horie, Seichi (Japan)

Horiguchi, Hyogo (Japan)

Ichihara, Gaku (Japan)

Idris, Mohd (Malaysia)

Igarashi, Yu (Japan)

Iida, Mako (Japan)

Ikeda, Atsuko (Japan)

Ikeda, Hiroki (Japan)

Ikegami, Kazunori (Japan)

Imai, Teppei (Japan)

Inoue, Akiomi (Japan)

Inoue, Koki (Japan)

Irigoyen‐Otiñano, María (Spain)

Ishimaru, Tomohiro (Japan)

Ishitake, Tatsuya (Japan)

Itani, Osamu (Japan)

Ito, Akiyoshi (Japan)

Ito, Yuki (Japan)

Iwakiri, Kazuyuki (Japan)

Iwasaki, Akio (Japan)

Iwasaki, Shinichi (Japan)

Iwasawa, Satoko (Japan)

Iwata, Hiroko (Japan)

Izumi, Hiroyuki (Japan)

Janwantanakul, Prawit (Thailand)

Jurisic, Vladimir (Serbia)

Kakamu, Takeyasu (Japan)

Kanamori, Satoru (Japan)

Kanda, Kanae (Japan)

Kang, Mo‐Yeol (Korea, the Republic of)

Kang, Young‐Joong (Korea, the Republic of)

Katayama, Akihiko (Japan)

Kawada, Michiko (Japan)

Kawakami, Tsuyoshi (Thailand)

Kawanami, Shoko (Japan)

Kawashima, Masatoshi (Japan)

Khalili, Arash (Iran)

Kido, Takamasa (Japan)

Kishi, Taro (Japan)

Kitamura, Hiroko (Japan)

Kodithuwakku Arachchige, Sachini (United States)

Kojima, Reiji (Japan)

Kojin, Hiroyuki (Japan)

Kostelac, Deni (Croatia)

Kozaki, Tomoaki (Japan)

Kubo, Tomohide (Japan)

Kubo, Yoshiko (Japan)

Kurosawa, Hajime (Japan)

Kuwahara, Keisuke (Japan)

Lee, Jihye (Korea, the Republic of)

Leff, Todd (United States)

Leocadio‐Miguel, Mario (United Kingdom)

Lin, Ro‐Ting (Taiwan)

Liu, Xinxin (Japan)

Loh, Ping Yeap (Japan)

López Gómez, María Andrée (Spain)

Lu, Dasheng (China)

Lu, Ming‐Lun (Taiwan)

Macabulos, Edmyr (Philippines)

Maeda, Eri (Japan)

Maeda, Shunta (Japan)

Mafune, Kosuke (Japan)

Maruyama, Takashi (Japan)

Massimi, Azzurra (Italy)

Masuda, Masashi (Japan)

Matsugaki, Ryutaro (Japan)

Matsumoto, Junko (Japan)

Matsumoto, Shun (Japan)

Matsuo, Tomoaki (Japan)

Mbare, Benta (Finland)

Meucci, Rodrigo (Brazil)

Michishita, Ryoma (Japan)

Miki, Akiko (Japan)

Minami, Kouichiro (Japan)

Miyamoto,Toshiaki (Japan)

Mori, Koji (Japan)

Mori, Mihoko (Japan)

Mori, Takahiro (Japan)

Morimoto, Yasuo (Japan)

Morioka, Ikuharu (Japan)

Morita, Yusaku (Japan)

Murakami, Haruka (Japan)

Murakami, Takahisa (Japan)

Muto, Go (Japan)

Nagano, Chikage (Japan)

Nagata, Masako (Japan)

Nagata, Tomohisa (Japan)

Naif, Moath (Cyprus)

Nakamura, Mieko (Japan)

Nakata, Yoshio (Japan)

Neupane, Subas (Finland)

Nishi, Kenichiro (Japan)

Nishihama, Yukiko (Japan)

Nogawa, Kazuhiro (Japan)

Nomura, Kyoko (Japan)

Ogami, Akira (Japan)

Ogata, Toru (Japan)

Ogawa, Masanori (Japan)

Ohta, Masanori (Japan)

Okawara, Makoto (Japan)

Ono‐Ogasawara, Mariko (Japan)

Ose, Solveig (Norway)

Ota, Atsuhiko (Japan)

Otsuka, Toshiaki (Japan)

Otsuka, Yasumasa (Japan)

Otsuka, Yuichiro (Japan)

Park, Chang (United States)

Picó‐Monllor, José Antonio (Spain)

Puttonen, Sampsa (Finland)

Ramacciati, Nicola (Italy)

Riediker, Michael (Switzerland)

Ruseski, Jane (United States)

Saijo, Yasuaki (Japan)

Sakai, Kosuke (Japan)

Sakakibara, Keiko (Japan)

Sakuraya, Asuka (Japan)

Sasaki, Minako (Japan)

Sasaki, Natsu (Japan)

Sato, Yukihiro (Japan)

Sawada, Shinichi (Japan)

Sawada, Susumu (Japan)

Schleupner, Ricarda (Austria)

Shibata, Eiji (Japan)

Shimazu, Akihito (Japan)

Shimura, Akiyoshi (Japan)

Shinada, Kayoko (Japan)

Sieber, W. Karl (United States)

Smith, Todd (United States)

So, Rina (Japan)

Song, Fujian (United Kingdom)

Sriram, Krishnan (United States)

Sugai, Toshiki (Japan)

Sugama, Atsushi (Japan)

Sugano, Ryosuke (Japan)

Sukadarin, Ezrin Hani (Malaysia)

Suzuki, Ayako (Japan)

Svensson, Thomas (Sweden)

Tachi, Norihide (Japan)

Tahara, Hiroyuki (Japan)

Takahara, Ryuji (Japan)

Takahashi, Toru (Japan)

Takahashi, Yukio (Japan)

Talapatra, Subrata (Bangladesh)

Tamakoshi, Koji (Japan)

Tani, Naomichi (Japan)

Tatsumi, Yukako (Japan)

Tokizawa, Ken (Japan)

Tominaga, Maki (Japan)

Tomita, Yoshihito (Japan)

Toyama, Hiroyuki (Finland)

Tsai, Feng‐jen (Taiwan)

Tse, Lap Ah (Hong Kong)

Tsuchiya, Masao (Japan)

Tsuda, Yoko (Japan)

Tsuji, Masayoshi (Japan)

Tsujimura, Hiroji (Japan)

Tsukinoki, Rumi (Japan)

Tsuno, Kanami (Japan)

Uchida, Mitsuo (Japan)

Ueta, Ikuo (Japan)

Ukawa, Shigekazu (Japan)

Van Wijk, Charles (South Africa)

Wada, Keiko (Japan)

Wada, Koji (Japan)

Watanabe, Kazuhiro (Japan)

Yamamoto, Makoto (Japan)

Yamauchi, Takenori (Japan)

Yatera, Kazuhiro (Japan)

Yokouchi, Nobutada (Japan)

Yoon, Jin‐Ha (Korea, the Republic of)

Yoshikawa, Etsuko (Japan)

Zaitsu, Masayoshi (Japan)

